# Single- and Multi-Joint Maximum Weight Lifting Relationship to Free-Fat Mass in Different Exercises for Upper- and Lower-Limbs in Well-Trained Male Young Adults

**DOI:** 10.3390/ijerph19074020

**Published:** 2022-03-28

**Authors:** Danilo A. Massini, Anderson G. Macedo, Tiago A. F. Almeida, Mário C. Espada, Fernando J. Santos, Eliane A. Castro, Daniel C. P. Ferreira, Cassiano M. Neiva, Dalton M. Pessôa Filho

**Affiliations:** 1 Graduate Programme in Human Development and Technology, São Paulo State University (UNESP), Rio Claro 13506-900, Brazil; dmassini@hotmail.com (D.A.M.); andersongmacedo@yahoo.com.br (A.G.M.); elianeaparecidacastro@gmail.com (E.A.C.); daniel.crisfe@unesp.br (D.C.P.F.); merussi.neiva@unesp.br (C.M.N.); dalton.pessoa-filho@unesp.br (D.M.P.F.); 2 Department of Physical Education, São Paulo State University (UNESP), Bauru 17033-360, Brazil; tiagofalmeida.w@gmail.com; 3 School of Education, Polytechnic Institute of Setúbal, 2914-504 Setubal, Portugal; fernando.santos@ese.ips.pt; 4 Life Quality Research Centre, 2040-413 Rio Maior, Portugal; 5 Faculty of Human Kinetics, University of Lisbon, 1499-002 Lisbon, Portugal; 6 LFE Research Group, Department of Health and Human Performance, Faculty of Physical Activity and Sport Sciences, Universidad Politécnica de Madrid (UPM), 28040 Madrid, Spain; 7 MEFE—Metabolism and Exercise Physiology Laboratory, Faculty of Science, São Paulo State University (UNESP), Bauru 17033-360, Brazil

**Keywords:** muscle strength, resistance exercise, body composition, early adulthood

## Abstract

This study aimed to analyze whether the relationship between regional and whole-body fat-free mass (FFM) and strength is related to FFM distribution and area according to limb involvement. Thirty well-trained male young adults underwent one-repetition maximum test (1RM) to assess the strength in arm curl (AC), bench press (BP), seated row (SR), leg press 45° (LP45), knee extension (KE), and leg curl (LC). Dual-energy X-ray absorptiometry was used to evaluate FFM. The values for 1RM in AC, BP, and R correlated to FFM in upper limb (R^2^ = 0.69, 0.84 and 0.75), without an effect of appendicular mass index (API) or area. For 1RM in KE, the correlation with FFM in lower limb increased with thigh area (R^2^ = 0.56), whereas 1RM in LC and LP45 correlation to whole-body FFM increased with API (R^2^ = 0.64 and 0.49). The upper limb’s FFM may be reliable for indexing the arms and upper trunk strengths, whereas the relationships between FFM and strength in lower limb improve as muscle mass and thigh area increases between subjects.

## 1. Introduction

Resistance exercise promotes muscular fitness (i.e., an increase in muscle strength and work economy, and improvement in power and speed during daily living or sporting tasks), which is undoubtedly accompanied by physiological and morphological muscle adaptations [1,2,3]. Nonetheless, muscle adaptation to resistance training requires that variables are planned (choice of exercise, order of exercise, load, volume, rest, frequency, and repetition velocity) to match a specific goal [2,4,5]. Indeed, when dealing with advanced practitioners (i.e., many years of training), further improvements in strength and muscle hypertrophy require the adequate management of training variables (e.g., load, repetition, sets, rest, and motor task) during a single session or throughout planning [1].

The loading in resistance training is operationally defined as the percentage of one-repetition maximum weight lifted (%1RM) in a single- or multi-joint exercise [4,5]. There are existing protocols for the measurement of the 1RM value [6]; however, these procedures are unreasonable when considering the training routine and planning for advanced practitioners, which include higher %1RM, high training volume (multiple sets, and a high number of repetitions), and high frequency to encompass a variety of single- and multi-joint exercises [1,5]. Alternatively, the monitoring of 1RM in terms of body composition and anthropometry is supported by the assumption that muscle strength increases in association with the modifications of fat-free mass (FFM) and, therefore, also influencing lift performance.

It was previously shown that segmental body area (arm circumference, arm muscle cross-sectional area, and thigh circumference) also makes a significant contribution to strength in highly resistance-trained athletes [7,8]. Moreover, the fewer joints and muscle groups involved in a weight lifting session, the greater the predictive accuracy from variables of body dimensions. However, the power of this relationship is controversial among studies [9,10]. Hortobágyi et al. [9] concluded that individual differences in muscular strength are poorly related to various measures of body size and segmental body dimensions, since correlations between strength vs. body mass, FFM, thigh and arm volume, cross-sectional area, and skinfolds ranged from −0.52 to 0.56 for trained and non-trained subject groups. Conversely, Hetzler et al. [10] evidenced improvements in the estimate of 1RM bench press using the repetitions to failure test with the addition of the arm circumference and arm length.

In the earliest studies reporting the relationship between 1RM values and anthropometric information, the coefficients widely ranged, but were not above 0.9 [8,11,12,13,14,15]. Therefore, when collectively analyzed, most of these previous studies have related sectional and muscle areas, circumference, and body mass to 1RM performance in multi-joint exercises (i.e., bench press and squat), resulting in predictive equations without the same robustness of the estimate as the models considering the submaximal level of muscle strength (i.e., repetition to failure based on a given weight, percentage of body mass, or fixed number of lifts) [16]. However, an improvement in correlation coefficient has been reported when FFM is considered as an independent variable to be related with the strength for exercises engaging single joints and small muscle groups [8,14] regardless of the level of training (i.e., moderate or advanced) of the participants [7,9,11,17].

Information is surprisingly lacking regarding the power of regional composition to monitor the 1RM value, despite findings indicating the influence of physical performance, FFM, and muscle fiber hypertrophy on the ability to lift heavier weight [18,19]. Indeed, if regional body tissue adaptations are considered to be meaningful information, combined with whole-body changes, and with practical (re)considerations for training control and planning across sexes and ages [20], it would be interesting to analyze how the regional composition information may be useful to evaluate the variations in 1RM in exercises regarding muscle mass participation in resistance exercises.

Thus, the objective of this study was to analyze whether regional and whole-body FFM, which are expected to correlate with 1RM in upper- and lower-limb exercises, follow a specific tendency concerning the limb engaged in exercise. In addition, we wondered whether regional FFM influences the change in 1RM values according to the differences in anthropometric and other composition variables between participants. We hypothesized that regional FFM correlation with 1RM values follows a specific trend regarding the limb engaged in the lift movement, therefore presenting a stronger coefficient compared to anthropometric and whole-body FFM variables. In others words, confirmation that strength and FFM are more strongly related at the body region level will demonstrate that muscle force and mass are both parameters of limb enhancement or a decreased ability in lifting exercises. This would support training and rehabilitation plans regarding body region requirements for strength improvements.

## 2. Materials and Methods

### 2.1. Participants

Thirty well-trained male adult volunteers (23.7 ± 5.8 years, 178.7 ± 5.3 cm in height, 78.7 ± 11.3 kg in body weight, and 17.0 ± 5.4% in body fat), with resistance training experience of at least two years and no injury episode during the last six months, provided their written informed consent to participate in this study. Only male young adults participated to avoid the interference of maturation, sex, and aging process on muscle strength, fat-free tissue mass, and bone mineral content among subjects [17,21]. This research was approved by the Local Ethics Committee of the University (CAEE: 19824719.3.0000.5398).

### 2.2. Body Composition

The dual-energy X-ray absorptiometry (DXA) method (Hologic^®^ model, QDR Discovery Wi^®^, Beldford, MA, USA) was used to obtain the regional and whole-body composition. The software (Hologic APEX^®^, Beldford, MA, USA) provided values of FFM (fat-free mass and bone mineral content, in grams) for upper and lower limbs (UL-FFM and LL-FFM), and the submaximal whole-body FFM (WB-FFM, discarding values for the head). Other regional and whole-body composition variables were fat mass (FM), area, and appendicular fat-free mass index (API). The equipment was calibrated following the manufacturer’s recommendations by a laboratory technician with experience in these procedures. According to Nana et al. [22], the standardized conditions for DXA scanning are: (i) participants be presented fasted, rested (no exercise), and with no fluid ingestion for at least three hours before the analysis, and (ii) should arrive wearing light clothing, without shoes or carrying any metallic object or body-worn accessories. During the DXA scanning, the participants remained lying in the supine position on the table until the end of the scan, with feet kept together (~15 cm apart) and arms arranged along the side of the trunk (in a mid-prone position with ~3 cm between the palms and trunk). The same technician adjusted the anatomical points following the manufacturer recommendations. The participants underwent DXA scanning during the first visit.

### 2.3. Strength Measurements

Tests of 1RM were performed on the following exercises: (1) arm curl (AC), (2) horizontal bench press (BP), (3) seated row (SR), (4) knee extension (KE), (5) leg curl (LC), and (6) leg press 45° (LP45). All tests were performed after a non-specific warm-up of 15 min (static stretching, cycling, or running at exercise intensity ≤60% age-predicted maximal heart rate (i.e., HRmax = 220 − age, with age in years). The protocol of the 1RM test followed the recommendations of Mayhew et al. [23]: (1) a specific warm-up preceded the first attempt of the test and was performed with light weights to avoid concentric failure, and up to 8–10 non-maximal repetitions; (2) initial test weight was chosen based on the average rates for the strength of upper- and lower-limbs, according to age, sex, and body mass [6]; and (3) participants performed at least three attempts of one repetition each, with three minutes of rest between each attempt. The weight was increased or decreased from the initial weight by 1.1 to 4.5 kg based on the difficulty of the first lift. The weight that could not be lifted twice (i.e., self-reported inability, or failure in attempt, to perform the second lift) represented the 1RM reference [6,7]. The load value was reported in kilograms (kg). The participants were instructed to perform the movements with the proper technique, following recommendations from Baechle and Earle [24]. Moreover, two visits, separated by 24 h, were scheduled for the completion of all 1RM testing, following the order of small to large muscle groups, intercalating upper- and lower-limb exercises. Thus, AC, KE, and BP were tested in the first visit, and LC, SR, and LP45 in the second visit. Participants were instructed to avoid high-intensity resistance training 48 h before the testing, and to present themselves rested, fasten, and well-hydrated two hours prior to testing.

### 2.4. Statistical Analysis

The data are reported as mean ± standard deviation, confidence interval (CI_95%_), and standard error of measurement (SEM). Normality was checked for the muscle strength variables by the Shapiro–Wilk test. The Pearson coefficient (r) was used to test the linear relationship (2-tailed) between maximum observed strength and body composition variables. The stepwise method was used to model the linear relationship between values of 1RM (as the dependent factor) and regional and whole-body composition variables (as independent factors). The input data for muscle strength in UL exercises considered regional and whole-body composition variables, except those for LL, and vice versa when the procedures were applied to analyze the relationship between LL strength exercises and body composition. To ensure that the correlations were not inflated for the differences in muscle area and musculature distribution, the analysis was controlled to segment area (i.e., arm or thigh, according to the body region involved in the exercise) and API (independently of the body region involved in the exercise). The Pearson coefficient was interpreted as <0.2 (trivial), 0.20–0.49 (small), 0.5–0.8 (medium), and >0.8 (strong) [24]. Scatterplots was used to analyze the explained variance (R^2^ _and_ R^2^_adj_) and standard error of the estimate (SEE) of the FFM-predicted 1RM distribution to the observed 1RM distribution across subjects, considering both coefficients as <0.04 (trivial), 0.04–0.24 (small), 0.25–0.63 (medium), and >0.64 (strong) [25]. All statistical procedures were performed in SPSS 26 (Statistical Package for Social Sciences, IBM, Armonk, NY, USA), with a significance level of *p* ≤ 0.05.

The sample power for the associations between the observed and predicted 1RM values were determined for each exercise, and the mean value was considered for analysis of the sample size (*n* = 30). Input parameters were: (a) the corresponding value of “*r*” from the coefficient for explained variance (R^2^) given in scatterplots; (b) *Zα* = 1.96 for a security index of *α* = 0.05, following Díaz and Fernandéz [26]:

(1) $$Z_{1-\beta }=\sqrt{n-3}\frac{1}{2}ln\left(\frac{1+r}{1-r}\right)-Z_{1-\frac{\alpha }{2}}$$

To avoid anon-realistic statistical power by using information from the actual sample, the cross-validation process was performed using the predicted residual error sum of squares (*PRESS*) method [27,28]. From the *PRESS* statistic, a modified form of R^2^ adjusted (R^2^_p_) and standard error of the estimate (SEE_p_) were recalculated, R^2^_p_ = 1 – (*PRESS*/SS_Total_) and SEE_p_ = (*PRESS*/*n*)^½^, in which *PRESS* is the sum of the squares of eliminated residuals:

(2) $$PRESS=\sum_{i=1}^{n}{\left(y_{i}-{\hat{y}}_{i,-i}\;\right)}^{2}$$

## 3. Results

Table 1 presents regional and whole-body composition characteristics, anthropometric area, and 1RM values of the participants. 

The correlation coefficients between the regional and whole-body composition variables with 1RM values are shown in Table 2. All correlation coefficients for UL- and LL-FFM were observed to be at a higher level than those for API, WB-FFM, and arm and thigh areas, with the exceptions of KE, LC, and LP45, for which the correlations with WB-FFM and LL-FFM were quite similar.

**Figure 1:**
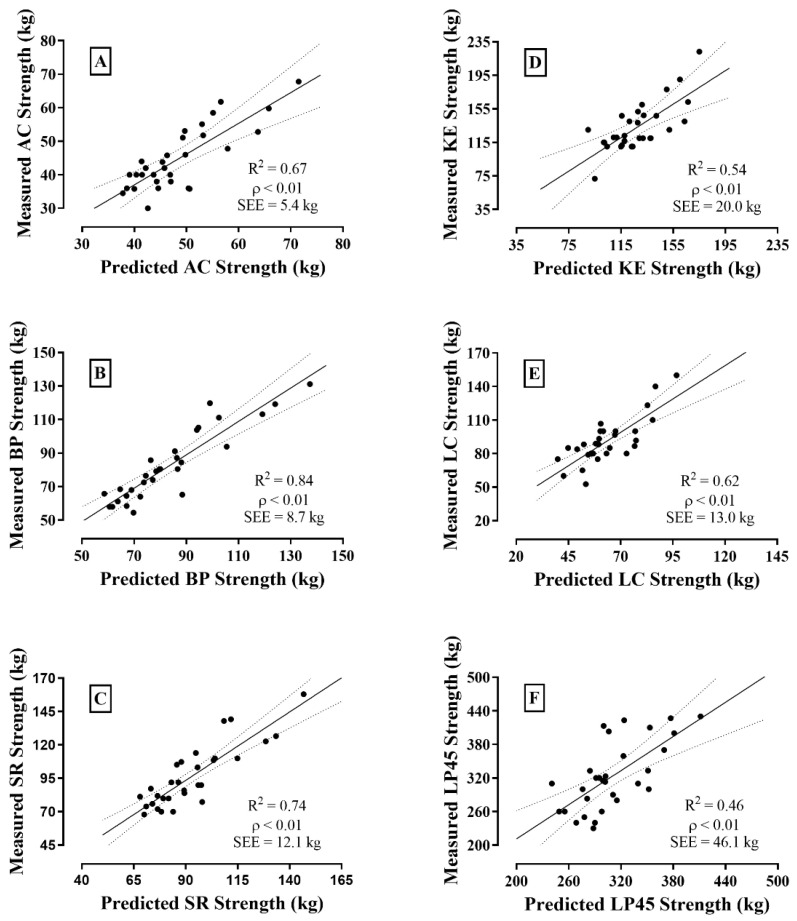
Scatterplots between observed and predicted values for the 1RM tests in bench press (BP) (**A**); arm curl (AC) (**B**); seated row (SR) (**C**); knee extension (KE) (**D**); leg curl (LC) (**E**); and leg press 45° (LP45) (**F**).

Figure 1 depicts the scatterplots between values for the 1RM tests. For AC, BP, and SR, the explained variances from UL-FFM (Figure 1A–C) were higher when controlled by API (R^2^ = 0.69, 0.84, and 0.75, respectively, (strong), *p* < 0.01). A similar result was observed for the KE variance explained by LL-FFM (Figure 1D), which increased when controlled by the thigh area (R^2^ = 0.54 (medium), *p* < 0.01), and for the LC and LP45 variances explained by WB-FFM (Figure 1E and F), which also increased when controlled by API (R^2^ = 0.62 (strong) and 0.46 (medium), respectively, *p* < 0.01).

The *PRESS* analysis is presented in Table 3. The stability of the correlations by shrinkage analysis from R^2^_adj_ to R^2^_p_ was ensured for all observed correlations between RE and FFM variables, since the values for R^2^_p_ were at a ≤0.1 ratio from the previous R^2^_adj_ values. Cross-validation was therefore acceptable from R^2^_p_ for regression analysis in all resistance exercises. Calculated unbiased estimates of SEE_p_ reduced when compared to those SEE shown in Figure 1 for all resistance exercises. 

## 4. Discussion

The aim of this study was to analyze whether regional and whole-body FFM follows a specific tendency concerning the limb engaged in exercise. In addition, we wondered whether regional FFM influences the change in 1RM values according to the differences in anthropometric and other composition variables between participants. The findings from the present study showed that both UL- and LL-FFM are powerful indexes that are related to 1RM measurements for single- or multi-joint resistance exercises engaging upper- and lower-limb actions. Therefore, our findings are aligned with the assumption that resistance training can improve muscle strength, weight lifting capacity, and fat-free body mass [2,18]. However, information on the propensity of regional body composition to analyze muscle strength variance in different weight lifting exercises is still lacking in the literature. Thus, the current study evidenced that 1RM correlations with FFM in upper and lower limbs in exercises involving single- and multi-joint actions increased according to the content of FFM, regardless of the peripheral FFM distribution and thigh area between subjects when considering resistance exercises involving upper and lower limbs (respectively).

In this sense, the way that FFM variables related with 1RM values for UL single- or multi-joint exercises evidenced a higher power for regional than whole-body FFM, regardless of the arm sectional area between subjects. Moreover, the LL-FFM is a relevant variable for 1RM values when considering LL lifting weight capacity. However, the LL-FFM variable did not achieve a higher power than the whole-body FFM for the correlation with all resistance exercises. The FFM peripheral distribution (i.e., API) accounted for the increase in the correlation coefficients for LC and LP45; therefore, the results suggest that the greater the engagement of muscle mass for the execution of the exercise, the less the regional influence of FFM seems to be. 

Undoubtedly, monitoring 1RM values based on regional FFM is an alternative way to control the muscle strength variation [8,29,30]. Moreover, a successful maximum lifted weight during a standard 1RM test protocol presumes: (i) movement expertise and engagement, (ii) soreness and injury possibilities, and (iii) changes in the weight lifted with the difference in mechanical demand of similar exercises. These are the greatest constraints for the testing protocol frequency and application to every exercise planned for training [29,30,31]. Therefore, the power of the interactions between maximum weight lifting capacity with body composition parameters (i.e., body mass, fat-free body mass, regional body area and volume, girth, and width) would provide confident references for 1RM measurements, controlling muscle strength improvements, and organizing or revising the overload during the training in accordance with the previous target weight and exercise volume [7,9,10,20,32,33,34].

However, the literature has shown conflicting results for assessing 1RM using anthropometric and body composition variables, mainly when it is carried out with subjects with differences in muscle strength. On the one hand, results showing that among trained subjects, anthropometric variables (arm circumference and length) improved the reliability (R^2^ changed from 0.87 to 0.90) of 1RM estimation in the bench press [10]. Additionally, the predictive power (multiple regression coefficient, R^2^) of the anthropometric dimension variables for 1RM estimates ranged from 0.52 to 0.87 for trained subjects [16,30]. Body composition and anthropometry have been related to variations in muscle strength among untrained subjects, but evidence of associations with 1RM were small to medium (Pearson’s coefficient ranging from 0.42 to 0.67), mainly for LL and UL multi-joint resistance exercises [9,13,14,16].

Furthermore, 75.7% of the strength assessed in the bench press by trained men can be explained by the variations in the cross-sectional area of the arm, BMI, and fat percentage, with a standard error of 12.1 kg in the prediction [11]. In addition, the strength in the bench press exercise, in populations of both sexes and varying strength levels, showed a high correlation with the variable lean mass (0.77), and moderate correlations with height (0.59), body weight (0.56), arm circumference (0.66), and chest circumference (0.60), although only the lean mass and submaximal load for 10RM estimated the bench press strength with 97.6% explanatory capacity [8].

Thus, the statement that highly trained athletes exhibit closer relationships between anthropometric dimensions and weight lifted, and probably, the fewer joints and muscle groups involved in a lift, the greater the predictive accuracy of maximum performance by structural proportions [8], remain theories about the association between training development and the responses in the body’s dimensions. The results from the current study agree with this statement. Furthermore, we extend this assertion to exercises involving UL, considering that the association was independent of arm area size, but increased with FFM distribution in the upper limb. Moreover, for LL exercises, the control of 1RM values should consider changes in whole-body FFM and its peripheral distribution between subjects.

The lack of research relating regional body composition to 1RM for single-joint resistance exercises, contrast to those analyzing whole-body composition, anthropometry, and sub-maximal lifted weight relationships to 1RM for multi-joint resistance exercises. For example, the estimate of 1RM from a sub-maximal performance at 5RM or 10RM, with R^2^ ranging from 0.96 to 0.99, and SEE lower than 6 and 24 kg, respectively, for bench press and leg press, has been widely accepted as the alternative reference to predicted maximal muscle strength [30]. However, even when relying on submaximal muscle strength scores to estimate 1RM, it is well recognized that the same intrinsic determinant, such as sex and training status, can alter the maximum number of repetitions performed at certain fractions of 1RM [20]. Moreover, each type of exercise prescribed in resistance training requires its specific 1RM reference, and submaximal equations were not available to predict 1RM in different single- or multi-joint resistance exercises. Indeed, athletes should not agree to participate in time-consuming test procedures, or non-specific weight lifting, as these may disrupt their training planning.

However, the lack of a comparable sample of subjects to perform cross-validation of the present relationships hindered a better emphasis of the power of regional and whole-body FFM to predict lifting abilities in single- and multi-joint exercises because reproducibility and sensitivity were not evaluated. Nevertheless, the sample power for correlation analysis was above 80%, which is satisfactory to prevent type II errors. Moreover, cross-validation by applying the *PRESS* approach yielded values for R^2^_p_ and SEE_p_ that were appropriate to strengthen the demonstrated correlations. In addition, the standardized 1RM protocol used in the current study may be a source of underestimation of the maximal strength during the attempt to attained the heaviest load in a single lifting [4]. Despite the possible underestimation of the actual maximal strength, this does not necessarily mean that a heavy load was not attained during the last lifting attempt, and the attained load was therefore ensured to be very close to the maximal one (i.e., >95% 1RM). Nonetheless, the results should, strictly, be applied to the management of 1RM values in subjects who met the following conditions: (a) expertise in the resistance exercise performance mode; (b) engagement in resistance training for at least two years; and (c) UL-FFM, WB-FFM, and arm cross-sectional area as adjustments to the observed correlation values.

## 5. Conclusions

The current findings evidenced the role of regional fat-free tissue for monitoring the muscle strength development in specific body regions. This demonstrated that regional FFM may be applied to parametrize muscle strength in different resistance exercises for upper and lower limbs, and would explain rates of 81% and 75% for single-joint exercises, respectively. As a suggestion to improve the reliance in these or other indices of regional and whole-body composition, future analysis should focus on how maximal weight lifting relates to fat-free tissue across randomized trials for both sexes, before and after intervention with resistance exercises planned for muscle strength improvements in single- and multi-joint exercises separately.

## Figures and Tables

**Table 1:** Values of regional and whole-body composition, and muscle strength.

		Mean ± SD	CI_95%_	SEM
Index	API (kg/m^2^)	9.67 ± 1.02	9.30–10.06	0.19
FFM	WB (g)	59,596.8 ± 6831.6	57,045.9–62,147.8	1247.3
	UL (g)	8439.0 ± 1387.8	7920.7–8957.2	253.4
	LL (g)	22,316.1 ± 3064.9	21,171.7–23,460.6	559.6
Areas	Arm (cm^2^)	480.1 ± 45.7	463.0–497.1	8.3
	Thigh (cm^2^)	837.9 ± 95.5	802.2–873.6	17.4
Exercises 1RM	AC (kg)	44.8 ± 9.3	41.3–48.3	1.7
	BP (kg)	82.5 ± 21.2	74.6–90.7	3.9
	SR (kg)	96.5 ± 23.2	87.9–105.2	4.2
	KE (kg)	133.4 ± 29.1	122.5–144.2	5.3
	LC (kg)	90.8 ± 20.7	83.1–98.6	3.8
	LP45 (kg)	323.6 ± 61.5	300.6–346.6	11.2

API: appendicular fat-free mass index; FFM: fat-free mass; WB: whole-body; UL upper limbs; LL: lower limbs; AC: arm curl; BP: horizontal bench press; SR: seated row; KE: knee extension; LC: leg curl; LP45: leg press 45°; 1RM: one-repetition maximum; SD: standard deviation; CI_95%_: confidence interval; SEM: standard error of measurement.

**Table 2:** Coefficients for Pearson’s correlation analysis between 1RM values and regional and whole-body composition variables.

Exercises	Body Composition					
	API				Area	
		WB-FFM	UL-FFM	LL-FFM	Arm	Thigh
AC	0.67 ** [medium]	0.71 ** [medium]	0.82 ** [strong]	na	0.60 ** [medium]	na
BP	0.83 ** [strong]	0.86 ** [strong]	0.91 ** [strong]	na	0.73 ** [medium]	na
SR	0.73 ** [medium]	0.83 ** [strong]	0.86 ** [strong]	na	0.76 ** [medium]	na
KE	0.56 ** [medium]	0.71 ** [medium]	na	0.74 ** [medium]	na	0.65 ** [medium]
LC	0.58 ** [medium]	0.79 ** [medium]	na	0.77 ** [medium]	na	0.72 ** [medium]
LP45	0.60 ** [medium]	0.68 ** [medium]	na	0.63 ** [medium]	na	0.50 ** [small]

API: appendicular fat-free mass index; WB: whole-body; FFM: fat-free mass; UL: upper limbs; LL: lower limbs; AC: arm curl; BP: horizontal bench press; SR: seated row; KE: knee extension; LC: leg curl; LP45: leg press 45°. ** *p* < 0.001, na: not analyzed.

**Table 3:** Cross-validation values from *PRESS* analysis.

Exercise	Model	Cross-Validation			
	R^2^_adj_	R^2^_p_	Shrinkage	SEE_p_ (kg)	SEE_Dif_ (%)
AC	0.66	0.63	0.03	5.54	+2.21
BP	0.83	0.82	0.01	8.92	+2.41
SR	0.73	0.70	0.03	12.44	+2.89
KE	0.53	0.43	0.01	21.56	+7.85
LC	0.61	0.55	0.06	13.71	+5.79
LP45	0.44	0.40	0.04	46.93	+1.89

AC: arm curl; BP: horizontal bench press; SR: seated row; KE: knee extension; LC: leg curl; LP45: leg press 45°; SEE: standard error of estimate. SEE_Dif_: difference between SEE_p_ and SEE.

## Data Availability

The data that support the findings of this study are available from the corresponding and last author (mario.espada@ese.ips.pt and dalton.pessoa-filho@unesp.br), upon reasonable request.
